# Effect of supplementing L-cysteine and its group analogs on frozen semen quality of bulls: A meta-analysis

**DOI:** 10.14202/vetworld.2022.2517-2524

**Published:** 2022-11-09

**Authors:** Sari Yanti Hayanti, Mohammad Miftakhus Sholikin, Anuraga Jayanegara, Mokhamad Fakhrul Ulum, Marchie Astrid da Costa, Fitriawaty Fitriawaty, Surya Surya, Maureen Chrisye Hadiatry, Santiananda Arta Asmarasari, Eko Handiwirawan, Yenny Nur Anggraeny, Eni Siti Rohaeni, Salfina Nurdin Ahmad, Bustami Bustami, Aryogi Aryogi, Dicky Pamungkas, Yenni Yusriani

**Affiliations:** 1 Research Center for Animal Husbandry, Research Organization for Agriculture and Food, National Research and Innovation Agency of The Republic of Indonesia (BRIN), Cibinong Sciences Center, Cibinong, Bogor 16911, Indonesia; 2 Animal Feed and Nutrition Modelling Research Group, Faculty of Animal Science, IPB University, Bogor 16680, West Java, Indonesia; 3 Department of Nutrition and Feed Technology, Faculty of Animal Science, IPB University, Bogor 16680, West Java, Indonesia; 4 Division of Veterinary Reproduction, Obstetrics, and Gynaecology, Department of Veterinary Clinic, Reproduction, and Pathology, School of Veterinary Medicine and Biomedical Sciences, IPB University, Bogor, 16680, West Java, Indonesia

**Keywords:** bull cattle, cryopreservation, L-cysteine, meta-analysis, sperm

## Abstract

**Background and Aim:**

The quality of frozen bull sperm after thawing is influenced by the primary diluent and antioxidant. This meta-analysis was conducted to determine the effect of supplementing L-cysteine and its group analogs on the quality of frozen bull sperm.

**Materials and Methods:**

A total of 22 articles obtained from Google Scholar and Scopus were integrated into metadata. The effects of adding L-cysteine and its analogs (e.g., cysteine HCl and N-acetyl-L-cysteine), both of which are known as L-cysteine, were evaluated in this meta-analysis. The following parameters were examined: Abnormality, acrosome damage, acrosomal integrity, DNA damage, DNA integrity, malondialdehyde (MDA) content, plasma membrane integrity, pregnancy rate, progressive motility, sperm viability, and total motility. Data were analyzed using the mixed model methodology, with L-cysteine dosage as a fixed effect and different studies as random effects.

**Results:**

L-cysteine supplementation significantly increased the total motility (p < 0.05) and MDA content of semen, following a linear pattern. Progressive motility, acrosomal integrity, and plasma membrane integrity were significantly increased, showing a quadratic pattern (p < 0.05). Abnormality and acrosome damage were significantly decreased (p < 0.05), following a quadratic and linear pattern, respectively. Other parameters remained unaffected by L-cysteine supplementation. L-cysteine and cysteine HCl significantly inhibited (p = 0.001) acrosome damage in thawed frozen sperm compared with control sperm.

**Conclusion:**

Supplementing L-cysteine and its analog groups are recommended for freezing bull semen as it generally improves sperm quality.

## Introduction

Artificial insemination (AI) with frozen sperm remains the preferred technology for increasing the number of cattle [1]. The AI allows for the propagation and dissemination of superior sire genetics, increases the rate of genetic improvement and production gains, allows superior sires to produce significantly more offspring than natural service, and even enables the use of sires that cannot reproduce naturally or are no longer alive [2]. The potential of AI has also been demonstrated in livestock [3] and wildlife conservation [4–6]. Multiple processes culminate in cryopreservation as the final step in producing frozen bull sperm [7]. Freezing bovine sperm cells can subject them to physical and chemical stress [8]. Damaged sperm cells are characterized by low plasma membrane integrity, acrosome and mitochondrial damage, and DNA damage, reducing their viability and motility [9].

A success factor for AI with frozen sperm is a small number of damaged sperm cells after thawing [10]. Numerous techniques, including the addition of antioxidants to frozen semen diluents, have been attempted to improve the quality of bull sperm [11]. L-cysteine is found in extracellular cells and is a member of the non-essential amino acid antioxidant group [12, 13]. It is a semi-essential amino acid that has some analogs, including cysteine HCl, L-cysteine, and N-acetyl-L-cysteine. L-cysteine inhibits the production of free radicals generated by sperm cell metabolism [14]. In studies on L-cysteine supplementation for frozen sperm diluents, there were differences in the breed of cattle, the macroscopic and microscopic characteristics of fresh sperm, the primary diluent, and the dosage of L-cysteine administered. Therefore, a systematic review is required to determine the linearity of the effect of L-cysteine administration on bull sperm diluent. A meta-analysis is a powerful technique for analyzing multiple studies using comparable variables [15].

Therefore, we conducted this meta-analysis to determine the effect of L-cysteine supplementation on the quality of frozen bovine sperm.

## Materials and Methods

### Ethical approval

This type of research does not require ethical approval.

### Study period and location

The research was conducted from March to June 2022 in Jambi Assessment Institute for Agricultural Technology.

### Metadata development

This meta-analysis was conducted according to the methodology outlined in the Preferred Reporting Items for Systematic Reviews and Meta-Analyses guidelines [16]. The data used in this meta-analysis were extracted from previously published articles and entered into a meta-data. Published articles were searched using Google Scholar (https://scholar.google.com/) and Scopus (https://www.scopus.com) using the search terms “L-Cysteine,” “Cysteine HCl,” “N-Acetyl-L-Cysteine,” “amino acid,” “antioxidant,” “bull,” “bovine,” “bull cattle,” “sperm,” “cryopreservation,” and “frozen.”

### Inclusion and exclusion criteria

The primary inclusion criteria were a journal article written in English and published by a reputable publisher, the experimental design must have complied with the correct statistical rules, the amount of experimental and replicated material should have met the correct statistical standards, the study must have explicitly used the experimental animal material for bulls, and the number of bulls used (n) must have met the correct statistical requirements. The inclusion and exclusion criteria of the articles are presented in Fugure-1.

**Figure-1:**
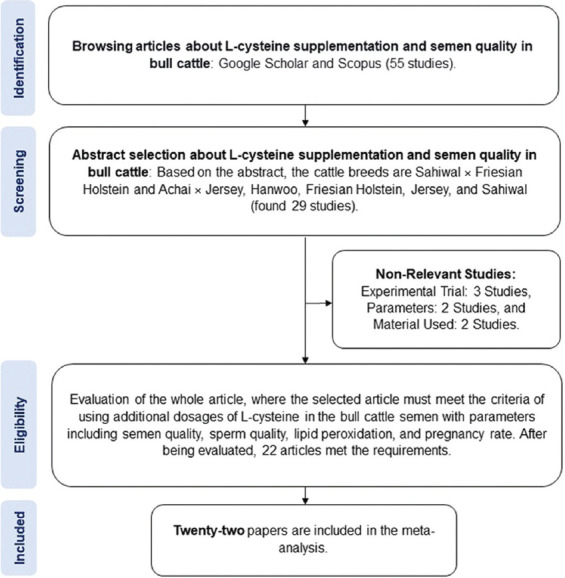
The selection of articles and evaluation process using the PRISMA method. PRISMA=Preferred Reporting Items for Systematic Reviews and Meta-Analyses.

### Data extraction

Author(s) names, publication year, journal name, breed of bulls, number of bulls, L-cysteine and its analog dosage, observed parameters, recommended extracted dosage, type of L-cysteine (e.g., cysteine HCl [T1], L-cysteine [T2], and N-acetyl-L-cysteine [T3]), units for each parameter, sampling technique, and parameter measurement technique were extracted from the selected articles. The search initially retrieved approximately 55 papers describing studies of L-cysteine supplementation for beef cattle. However, based on their titles and abstracts, only 29 of these papers had the potential to be included.

After a comprehensive evaluation, 22 articles were included in the metadata (Table-1). The selected papers included 11 beef and 11 dairy cattle studies [14, 17–37]. According to the available research, sperm is collected from sexually mature bulls. Abnormality (%), acrosome damage (%), acrosomal integrity (%), DNA damage (%), DNA integrity (%), malondialdehyde (MDA) content (nmol/mL), plasma membrane integrity (%), pregnancy rate (%), progressive motility (%), sperm viability (%), and total motility (%) were some of the performance parameters evaluated to examine the effect of L-cysteine supplementation. The metadata were updated with L-cysteine dosage. No unit conversion was performed on the data in this study because no different units were found. Descriptive statistical analysis was conducted to determine the data distribution (Table-2).

**Table-11:** Studies included in the meta-analysis.

Study	Reference	Cattle breed	Reported parameters	L-cysteine		

				Type	Dosage (mM)	Re.
1	[17]	Sahiwal	ACI and PMI	T2	0, 0.5, 1, and 2	2
2	[18]	Jersey	ACI, PGM, and TTM	T1	0, 10, 12, and 15	10
3	[19]	Friesian Holstein	PGM and TTM	T2 and T3	0, 0.5, and 1	1
4	[20]	Friesian Holstein	ABN, ACD, MDA, PGM, PRR, and TTM	T2	0 and 5	5
5	[21]	Bull	ABN, ACD, PRR, SPV, and TTM	T2	2 and 10	10
6	[22]	Friesian Holstein	ABN, PGM, and PMI	T2	0 and 1	1
7	[23]	Sahiwal×Achai and Friesian Holstein×Jersey	ACI, DNI, PGM, PMI, SPV, and TTM	T2	0, 5, 7.5, and 10	7.5
8	[24]	Hanwoo	ACD and SPV	T2	0 and 10	10
9	[25]	Hanwoo	ACD and SPV	T2	0, 5, 10 and 20	20
10	[26]	Bull	DND, PMI, and SPV	T3	0, 0.01, 0.1, 1, and 10	10
11	[27]	Jersey	ABN, ACD, DND, PMI, PRR, and SPV	T2	0 and 5	5
12	[28]	Jersey	ABN, ACD, PMI, SPV, and TTM	T1	0 and 5	5
13	[29]	Jersey	ACD, PGM, PMI, PRR, and TTM	T1	0 and 5	5
14	[30]	Friesian Holstein	DNI and PMI	T2	0 and 10	10
15	[31]	Friesian Holstein	DNI, PMI, SPV, and TTM	T2	0, 3, 5, and 7	5
16	[32]	Friesian Holstein	ABN, ACD, DND, PMI, PRR, and TTM	T2	0 and 2	2
17	[33]	Friesian Holstein	ABN, ACD, PMI, MDA, PRR, and TTM	T2	0 and 2	2
18	[34]	Friesian Holstein	ABN, ACD, and DNS	T2	0 and 5	5
19	[14]	Friesian Holstein	ABN, ACD, DND, MDA, PMI, PRR, and TTM	T2	5 and 10	10
20	[35]	Friesian Holstein	ACD, DND, and PRR	T2	0 and 2.5	2.5
21	[36]	Friesian Holstein	ABN, ACD, PMI, and TTM	T2	0 and 5	5
22	[37]	Achai and Friesian Holstein	ACI, PGM, PMI, and SPV	T2	0, 0.5, 1, and 1.5	1.5

ABN=Abnormality, ACD=Acrosome damage, ACI=Acrosomal integrity, DND=DNA damage, DNI=DNA integrity, MDA=Malondialdehyde, PMI=Plasma membrane integrity, PRR=Pregnancy rate, PGM=Progressive motility, Re.=Recommendation, SPV=Sperm viability, T1=Cysteine HCl, T2=L-cysteine, T3=N-acetyl-L-cysteine, TTM=Total motility

**Table-2:** Statistical descriptive analysis of the meta-analysis data.

Variable	N	Mean	SE	Max	Min
L-cysteine					
Dosage, mM	81	3.27	0.47	20	0
Parameter					
Abnormality, %	34	13.3	228	23	4.8
Acrosome damage, %	36	8.98	0.72	21.5	0.89
Acrosomal integrity, %	24	58.8	1.34	78.2	40.2
DNA damage, %	15	12.1	2.44	57	0.84
DNA integrity, %	14	94.5	1.4	100	62.6
Malondialdehyde, nmol/mL	12	2.55	4.61	5.96	0.7
Plasma membrane integrity, %	53	54.1	2.58	76.8	27.8
Pregnancy rate, %	24	51.5	2.58	81.5	15
Progressive motility, %	46	32.4	0.84	63.9	9.43
Sperm viability, %	49	62.4	0.63	79.3	40.8
Total motility, %	50	55.1	0	81.4	38.8

N=Number of data, MAX=Maximum value of the variables, Min=Minimum value of the variables, SE=Standard error

### Statistical analysis

Data analysis was conducted using the R software, version 4.2.0 (https://cran.r-project.org/), along with supplementary packages, including “data. table,” “DescTools,” “lme4,” “lmerTest,” “lsmeans,” “multcomp,” “multcompView,” “openxlsx,” “performance,” “readxl,” “reshape2,” and “tidyverse” [38–40]. The linear mixed model methodology was used for this meta-analysis. Random effects were used in this study, and the added dosage of L-cysteine was considered as a fixed effect [41–43]. The following equation will guide you through the mathematical model.


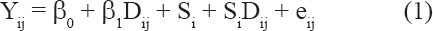



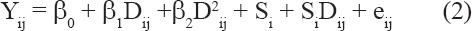


Where, (1) linear mixed model of the 1^st^ order, (2) linear mixed model of the 2^nd^ order, Y_ij_ was the dependent variable, β_0_ was the overall intercept across all studies (fixed effect), annotation 1 in β was the linear regression coefficient of Y on D (fixed effect) else was the quadratic regression coefficient of Y on D (fixed effect), D_ij_ was the value of the continuous predictor variable (addition of L-cysteine dosage), S_i_ was the random effect of study (i-th), and S_i_D_ij_ was to evaluate the appropriateness of statistical models, the p-value, root mean square error (RMSE), and R square Nakagawa were used [41, 44, 45]. If p ≤ 0.05, this indicated that the result was statistically significant. In addition to this, there was a tendency for the result to be significant if the p-value was only in the range of 0.05–0.1. To compare different types of L-cysteine, such as cysteine HCl, L-cysteine, and N-acetyl-L-cysteine, the least squares mean was used. Least mean squares were also used to evaluate the effects of bull breeds on sperm characteristics.

## Results

The relationship between the addition of L-cysteine and post-thawing frozen bovine sperm quality is shown in Table-3. The dosage of L-cysteine added to frozen bovine semen exerted a linear and quadratic effect on post-thawing sperm quality. It exerted a linear effect on total motility, sperm viability, DNA integrity, DNA damage, acrosome damage, MDA content, and pregnancy rate and a quadratic effect on progressive motility, abnormality, acrosomal integrity, and plasma membrane integrity. The dosage and type of L-cysteine showed an interaction with sperm viability (p = 0.031). However, the other parameters showed no significant interaction between dosage × type, dosage × breed of cattle, and type × breed of cattle.

**Table-3:** Effect of L-cysteine dosage (mM) on the quality of frozen bull sperm.

Parameter	N	M	β_0_		β_1_ and β_2_		Model validation			Interaction		
		
			Value	SE	Value	SE	p-value	RMSE	R^2^	D×C	D×B	C×B
Abnormality, %	34	Q	14.9	0.985	−1.34	0.359	0.002	2.37	0.56		0.078	0.308
					0.118	0.0312	0.001					
Acrosome damage, %	36	L	9.27	1.52	−0.25	0.075	0.004	1.11	0.94		0.781	0.409
Acrosomal integrity, %	24	Q	56.4	5.95	3.21	1.41	0.036	6.25	0.77		0.698	0.956
					−0.279	0.112	0.024					
DNA damage, %	15	L	16.1	8.5	−0.413	0.241	0.124	1.92	0.98		0.85	
DNA integrity, %	14	L	88.3	4.76	1.03	0.554	0.096	6.39	0.5		0.051	0.838
Malondialdehyde, nmol/mL	12	L	1.43	0.496	0.362	0.083	0.003	0.525	0.81			
Plasma membrane integrity, %	53	Q	51.9	2.52	2.11	0.943	0.032	5.24	0.68		0.475	0.956
					−0.23	0.104	0.033					
Pregnancy rate, %	24	L	49	4.81	0.869	0.6	0.173	5.23	0.81		0.471	
Progressive motility, %	46	Q	29.4	4.5	1.88	0.658	0.008	3.98	0.92	0.311	0.238	0.993
					−0.184	0.058	0.004					
Sperm viability, %	49	L	61.5	2.22	0.13	0.233	0.58	5.57	0.53		0.031	0.299
Total motility, %	50	L	53.5	2.27	0.402	0.189	0.043	2.64	0.89	0.32	0.761	0.982

B=Breed of bulls, C=Type of L-cysteine, D=L-cysteine dosage (mM), L=Linear, Q=Quadratic, M=Model, N=Number of data, RMSE=Root means square error, SE=Standard error

The dosage of L-cysteine exerted a positive significant effect (p < 0.05) on abnormality (p-linear (pl) = 0.002 and p-quadratic (pq) = 0.001), acrosome damage (p = 0.004), acrosomal integrity (pl = 0.036 and pq = 0.024), plasma membrane integrity (p < 0.05), progressive motility (p < 0.05), MDA content (p < 0.05), and total motility (p = 0.043). However, the dosage of L-cysteine exerted no significant effect on DNA damage (p = 0.124), DNA integrity (p = 6.39), pregnancy rate (p = 1.73), and sperm viability (p = 0.58). The DNA integrity, MDA content, plasma membrane integrity, pregnancy rate, progressive motility, sperm viability, and total motility were increased, but the abnormality, acrosome damage, and DNA damage of frozen bull sperm cells were suppressed by the dosage of L-cysteine.

The relationship between the type of L-cysteine and the quality of post-thawing frozen bovine semen is presented in Table-4. L-cysteine types exerted a different effect (p = 0.001) on the acrosome damage of post-thawing frozen semen. L-cysteine and cysteine HCl (7.6% and 6.8%, respectively) exerted a lower effect than the control (9.7%). However, the various cysteine types exerted a significantly higher effect (p = 0.003) on the total motility of post-thawing frozen semen in the following increasing order: L-cysteine (53%), N-acetate-L-cysteine (59%), and cysteine HCl (68.5%) and, in contrast, the type of L-cysteine exerted no different effect (p > 0.05) on abnormality (p = 0.132), acrosomal integrity (p = 0.612), DNA damage (p = 0.39), DNA integrity (p = 0.108), MDA content (p = 0.081), plasma membrane integrity (p = 0.107), pregnancy rate (p = 0.63), progressive motility (p = 0.438), and sperm viability (p = 0.057).

**Table-4:** The effects of various types of L-cysteine (cysteine HCl, L-cysteine, and N-acetyl-L-cysteine) on the quality of frozen bull sperm.

Parameter	p-value	Control	T1	T2	T3
Abnormality, %	0.132	14.8	14	11.9	
Acrosome damage, %	0.001	9.7^b^	6.8^a^	7.6^a^	
Acrosomal integrity, %	0.612	55.8	58	60.2	
DNA damage, %	0.39	16.4		13.4	16
DNA integrity, %	0.108	87.7		96.5	
Malondialdehyde, nmol/mL	0.081	1.7		3.2	
Plasma membrane integrity, %	0.107	51.4	59.8	53.5	63.7
Pregnancy rate, %	0.63	50.1	58.9	52	
Progressive motility, %	0.438	28.9	30.5	32.2	32.6
Sperm viability, %	0.057	58.4	59.7	64	67.4
Total motility, %	0.003	50.9^a^	68.5^b^	53.3^a^	59^ab^

T1=Cysteine HCl, T2=L-cysteine, T3=N-acetyl-L-cysteine. The error rate for superscript variations on the same line is 5%

The effect of bull breeds on the characteristics of frozen sperm is shown in Table-5. Compared with Hanwoo (18.2%) and Jersey (13.4%) bulls, the acrosome damage in Friesian Holstein bull sperm was comparatively less (4.1%). The Jersey bull showed the maximum plasma membrane integrity among all breeds (63.1%). The plasma membrane integrity values in the maximum-to-minimum order are as follows: Jersey > Friesian Holstein > Achai > Sahiwal > Friesian Holstein × Jersey > Sahiwal × Achai. The pregnancy rate of Friesian Holstein breed was substantially higher (p = 0.03) than that of Jersey breed. The effect of bull breed on other sperm characteristics was not statistically significant.

**Table-5:** The effect of bull breeds on the characteristics of frozen sperm.

Parameter	p-value	B_1_	B_2_	B_3_	B_4_	B_5_	B_6_	B_7_
Abnormality, %	0.55		13			14.2		
Acrosome damage, %	<0.001		4.1^a^		18.2^b^	13.4^b^		
DNA damage, %	0.86		19.3			15.2		
Plasma membrane integrity, %	0.08	50.7^ab^	47.6^a^	52.5^ab^		63.1^b^	47.7^ab^	46.5^ab^
Pregnancy rate, %	0.03		54.6^b^			32.7^a^		
Progressive motility, %	0.14	31.2	26.2	20.4		57.1	49.6	17.6
Sperm viability, %	0.4	70.6	69.3	59.6	61.2	60.1	66.8	54.9
Total motility, %	0.2		52.1	57.2		63.7		53.6

B_1_=Achai, B_2_=Friesian Holstein, B_3_=Friesian Holstein × Jersey, B_4_=Hanwoo, B_5_=Jersey, B_6_=Sahiwal, B_7_=Sahiwal × Achai. The error rate for superscript variations on the same line is 5%

The following mathematical equation may be derived from Table-3:


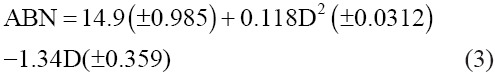



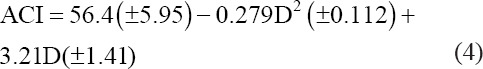



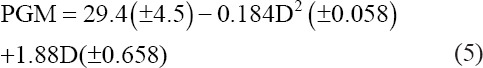



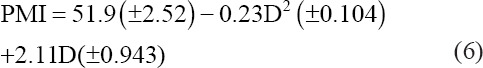


Where, ABN is abnormality, ACI is acrosomal integrity, D is dosage of L-cysteine (and its analogs), PGM is progressive motility, and PMI is plasma membrane integrity. Equations 3–6 are quadratic equations that have been identified from the analysis of the effect of L-cysteine dosage on abnormality (3), acrosomal integrity (4), progressive motility (5), and plasma membrane integrity (6). This quadratic form is useful for determining the optimum dosage of treatment by decreasing the quadratic function


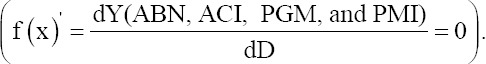


Then, the optimum value of L-cysteine dosage was obtained and the prediction of the optimal value of the parameters sequentially as follows: Abnormality (D = 5.69 mM, Y = 11%); acrosomal integrity (D = 5.76 mM, Y = 65.6%); progressive motility (D = 5.11 mM, Y = 34.2%); and plasma membrane integrity (D = 4.59 mM, Y = 56.8%).

## Discussion

Seminal fluid plasma in sperm consists of multiple antioxidants [46] and amino acids that positively correlate with sperm viability [47]. Frozen sperm contains lower levels of glutathione peroxidase, superoxide dismutase, reduced glutathione (GSH), and oxidized glutathione (GSSG) than fresh sperm [48].

The change in semen temperature from freezing to thawing affects the production of reactive oxygen species (ROS), increasing the amount of MDA [49]. The MDA is used to determine the level of ROS in bovine sperm [50]. According to Perumal *et al*. [29], the addition of L-cysteine can inhibit the production of lipid peroxidation, that is, MDA levels, resulting in a low ROS concentration. This finding is consistent with a meta-analysis that demonstrated that the administration of the antioxidant L-cysteine could inhibit MDA production in frozen bovine sperm after thawing.

Previous studies have shown that after thawing, the level of ROS increased in frozen bovine sperm, diminishing their viability and motility [37, 51]. In some buffaloes and sheep, the administration of L-cysteine inhibited the decrease in glutathione levels in bovine sperm, similar to that in cattle [27, 52, 53]. L-cysteine functions to reduce ROS levels by boosting glutathione profiles [48]. L-cysteine is produced by reduced L-cystine after it has traversed the cell membrane [54]. As a precursor for protein synthesis and GSH production, intracellular L-cysteine is essential for cellular homeostasis [12].

The addition of L-cysteine to semen exerted a significant effect (p < 0.05) in reducing the proportion of abnormal sperm and damaged acrosomes in the post-thawed frozen semen of bulls (Table-1). These findings are consistent with the previous studies [18, 23]. Low sperm count and damaged acrosomes obstruct the enhancement of other sperm quality parameters. In terms of motility, progressive motility, acrosomal integrity, and plasma membrane integrity, the addition of L-cysteine enhanced the microscopic quality of sperm. These findings are agree with studies showing that L-cysteine administration prevents the decrease in the percentage of total motility, progressive motility, acrosomal integrity, and plasma membrane integrity [17, 24, 28, 33].

The optimal dosage of L-cysteine required to suppress abnormal sperm count was 5.59 mM, assuming that the minimum abnormal sperm proportion was 11%. The optimal dosage of L-cysteine required for acrosomal integrity is 5.67 mM, with a predicted acrosomal integrity value of 65.6%. To increase progressive motility to 34.2%, the required dosage of L-cysteine is 5.11 mM. Similarly, to improve the sperm plasma membrane integrity to 56.8%, the required L-cysteine dosage was 4.59 mM. The effectiveness of L-cysteine dosage on these four parameters ranges from an equal dosage of approximately 5 mM for optimizing each parameter that determines sperm quality.

Using computer-assisted sperm analysis, it was determined that the quality of fresh sperm obtained directly from males was superior to that of frozen sperm after thawing [55]. The process of dilution and freezing affects sperm quality during the production of frozen bovine semen [56, 57]. Temperature changes during the thawing process exert an effect on sperm quality, including the acrosome, during the stage of using frozen bovine semen [58, 59]. Therefore, antioxidant supplements are required to prevent organelle damage in sperm cells [60]. This meta-analysis demonstrated that the addition of the antioxidant L-cysteine could reduce acrosome damage.

## Conclusion

The addition of L-cysteine during the process of frozen semen production could maintain sperm quality after thawing. Therefore, we recommend L-cysteine as an important supplement for sperm cells. The optimal L-cysteine dosages recommended for addition to the diluent during semen processing were 4.58–5.75 mM to increase progressive motility, acrosome integrity, and plasma membrane integrity and 5.68 mM for reducing abnormalities.

## Authors’ Contributions

SYH: Conceptual and research design. MAC, FF, SS, and MCH: Searched the literature and collected. SYH and SAA: Literature selection and processed the data. MMS: Analyzed the data. SYH and MMS: Wrote the original draft. MMS, AJ, MFU, SYH, EH, YNA, ESR, SNA, BB, AA, DP, and YY: Supervised the research. AJ, MFU, EH, YNA, ESR, SNA, BB, AA, DP, and YY: Critical review of the manuscript. All authors have read and approved the final manuscript.
